# cGMP-independent nitric oxide signaling and regulation of the cell cycle

**DOI:** 10.1186/1471-2164-6-151

**Published:** 2005-11-03

**Authors:** Xiaolin Cui, Jianhua Zhang, Penglin Ma, Daniela E Myers, Ilana G Goldberg, Kelly J Sittler, Jennifer J Barb, Peter J Munson, Ana del Pilar Cintron, J Philip McCoy, Shuibang Wang, Robert L Danner

**Affiliations:** 1 Critical Care Medicine Department, Clinical Center, National Institutes of Health, Bethesda, Maryland, USA; 2 Mathematical and Statistical Computing Laboratory, Division of Computational Bioscience, Center for Information Technology, National Institutes of Health, Bethesda, Maryland, USA; 3 Flow Cytometry Core Facility, National Heart, Lung, and Blood Institute, National Institutes of Health, Bethesda, Maryland, USA; 4 Intensive Care Unit of the Military 309th Hospital, Haidian District of Beijing, People's Republic of China

## Abstract

**Background:**

Regulatory functions of nitric oxide (NO^•^) that bypass the second messenger cGMP are incompletely understood. Here, cGMP-independent effects of NO^• ^on gene expression were globally examined in U937 cells, a human monoblastoid line that constitutively lacks soluble guanylate cyclase. Differentiated U937 cells (>80% in G0/G1) were exposed to S-nitrosoglutathione, a NO^• ^donor, or glutathione alone (control) for 6 h without or with dibutyryl-cAMP (Bt_2_cAMP), and then harvested to extract total RNA for microarray analysis. Bt_2_cAMP was used to block signaling attributable to NO^•^-induced decreases in cAMP.

**Results:**

NO^• ^regulated 110 transcripts that annotated disproportionately to the cell cycle and cell proliferation (47/110, 43%) and more frequently than expected contained AU-rich, post-transcriptional regulatory elements (ARE). Bt_2_cAMP regulated 106 genes; cell cycle gene enrichment did not reach significance. Like NO^•^, Bt_2_cAMP was associated with ARE-containing transcripts. A comparison of NO^• ^and Bt_2_cAMP effects showed that NO^• ^regulation of cell cycle genes was independent of its ability to interfere with cAMP signaling. Cell cycle genes induced by NO^• ^annotated to G1/S (7/8) and included E2F1 and p21/Waf1/Cip1; 6 of these 7 were E2F target genes involved in G1/S transition. Repressed genes were G2/M associated (24/27); 8 of 27 were known targets of p21. E2F1 mRNA and protein were increased by NO^•^, as was E2F1 binding to E2F promoter elements. NO^• ^activated p38 MAPK, stabilizing p21 mRNA (an ARE-containing transcript) and increasing p21 protein; this increased protein binding to CDE/CHR promoter sites of p21 target genes, repressing key G2/M phase genes, and increasing the proportion of cells in G2/M.

**Conclusion:**

NO^• ^coordinates a highly integrated program of cell cycle arrest that regulates a large number of genes, but does not require signaling through cGMP. In humans, antiproliferative effects of NO^• ^may rely substantially on cGMP-independent mechanisms. Stress kinase signaling and alterations in mRNA stability appear to be major pathways by which NO^• ^regulates the transcriptome.

## Background

Nitric oxide (NO^•^) plays a pivotal role in vascular biology through both cGMP-dependent and -independent mechanisms. In health, NO^• ^regulates vascular tone by activating soluble guanylate cyclase [1-3]. However, other important effects of NO^• ^in the vasculature such as cytoprotection and anti-adhesion appear to occur independent of cGMP signaling [4-6]. Likewise, NO^• ^regulation of inflammation has frequently been associated with signal transduction events that do not involve cGMP [7,8]. NO^• ^induces TNFα in human cells by decreasing intracellular levels of cAMP, thereby removing cAMP-mediated repression of the TNFα promoter through a proximal Sp element [9,10]. Analogs of cAMP and Sp site mutation both block, while antagonists of cAMP-dependent protein kinase simulate the effect of NO^• ^on TNFα. [9,11]. In contrast to TNFα, NO^• ^induces interleukin-8 (IL-8) [12] through a distinct post-transcriptional mechanism that is both cGMP- and cAMP-independent. IL-8 mRNA is stabilized by NO^• ^activation of p38 MAPK, increasing its half-life and translation [13]. These and other reports [14-16]. suggest that cGMP-independent gene regulation by NO^• ^occurs through multiple pathways.

Similar to the regulation of blood pressure and inflammatory responses, NO^• ^regulation of cell proliferation is of central importance to circulatory health. Failure of this regulatory pathway has been linked to atherosclerosis and other forms of vascular dysfunction [17-19]. Despite extensive investigation, the relative contribution of cGMP-independent NO^• ^signaling in the regulation of cell cycle genes remains controversial. In rats, NO^• ^has been shown to activate transcription through cGMP-dependent effects on AP-1 promoter sites [20]. Also in rodents, a NO^•^-cGMP-PKA-ERK1/2 signal transduction pathway has been described that inhibits cell proliferation [21,22] and increases expression of p21/Waf1/Cip1 [23,24]. A master regulatory gene, p21 directly inhibits Cdk complexes [25,26] and represses the transcription of many cell cycle genes through CDE/CHR (cell cycle dependent element/cell cycle gene homology region) promoter elements [27,28]. In contrast to rodents, NO^• ^regulation of cell cycle genes in humans, including regulation of p21, appears to occur, at least in part, independent of cGMP [19,29]. However, a global examination of cGMP-independent NO^• ^effects on the transcriptome in general or on cell cycle genes specifically has not been undertaken in either rodents or humans.

Here, oligonucleotide microarrays and human U937 cells that lack soluble guanylate cyclase [9,30] were used to globally characterize the cGMP-independent effects of NO^• ^on gene expression. Differentiation with PMA was employed to render cells capable of cytokine production [9]. This treatment also forced >80% of cells into the G0/G1 phase of the cell cycle, which facilitated the analysis of cell cycle gene regulation. Since NO^• ^lowers cAMP levels in U937 cells [9] and cAMP is known to affect cell proliferation, NO^• ^effects were also tested in the absence and presence of a cell permeable cAMP analog. For genes affected by NO^•^-induced decreases in cAMP, cAMP analog would be expected to produce an apposite effect. Hypotheses generated from microarray results were further investigated by examining downstream changes in protein expression and signal transduction pathways.

## Results

### Functional distribution of NO^•^-regulated genes and hypothesis generation

**Figure 1:**
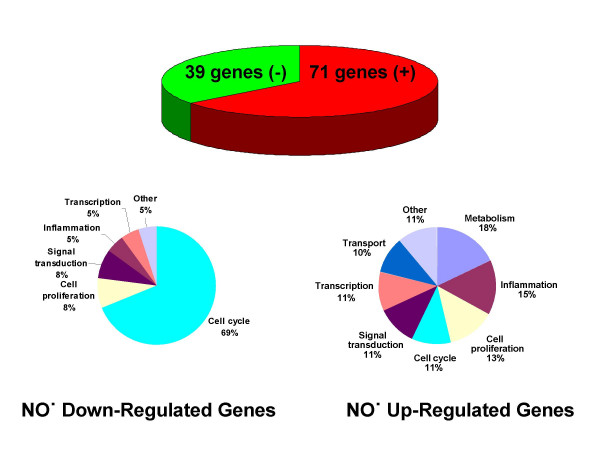
**Distribution of NO^•^-regulated genes. **Of 110 differentially regulated genes, 71 were up-regulated (red) and 39 (green) were down-regulated. Genes were classified into functional categories using NIH-DAVID [83] and PubMed [52]. Data are from seven independent microarray experiments.

Of 110 NO^•^-responsive genes, 71 were induced, and 39 were repressed; the majority were not previously known to be NO^•^-responsive. Both naïve and differentiated U937 cells lack NO^•^-sensitive soluble guanylate cyclase [9,30], and therefore gene regulation by NO^• ^in these cells can be attributed to cGMP-independent mechanisms. Genes were annotated into functional categories (Fig. 1) [see Additional files 1 and 2 for complete gene lists]. NO^• ^had broad biological effects independent of cGMP. Heme oxygenase 1 *(HMOX1)*, a known NO^•^-responsive gene, had the second largest fold change among up-regulated genes. TNFα and IL-8, cytokines previously associated with specific cGMP-independent mechanisms of NO^• ^regulation [8,9,12], were also detected as differentially regulated by the microarray analysis. NO^•^-regulated genes annotated disproportionately to the cell cycle [[31] of 106 (29%) compared to 407 of 4870 genes (8%) on the microarray, as annotated in the Gene Ontology [GO] Biological Process database; *P *= 0.0001] (Table 1). In particular, a large majority of NO^• ^down-regulated genes annotated specifically to the cell cycle (21/38, as annotated in the GO Biological Process database; *P *= 0.0001). Additional annotation using PubMed identified 47 of 110 genes (43%) as cell cycle or cell proliferation related. Of 39 down-regulated transcripts, 27 (69%) were ultimately annotated specifically to the cell cycle.

Previously, we demonstrated that NO^• ^activates p38 MAPK and thereby stabilizes IL-8 mRNA through effects on AU-rich elements [ARE] in 3' untranslated regions [UTR] [13]. Therefore, the ARE database  was used to identify ARE-containing genes among those regulated by NO^•^. Twenty-two of 110 genes contained ARE (20%) compared to 540 ARE genes of 5086 on the microarray (11%; *P *= 0.008). An additional 11 ARE-containing genes were identified in PubMed for a total of 33 (Table 2). Nearly half of these genes (14/33; 42%) have been reported to be p38 MAPK regulated (Table 2). Importantly, for these 14 genes, p38 MAPK activation produces responses that are in the same direction as those observed here for NO^•^. The broad influence of NO^• ^on cell cycle-related genes and ARE-containing transcripts independent of cGMP was unexpected, as was the strong association of these effects with p38 MAPK. Therefore, further experiments were performed to confirm these results and to define underlying regulatory mechanisms that might link NO^• ^effects on the cell cycle with post-transcriptional gene regulation through ARE sites.

**Table 2:** NO^•^-regulated genes containing AU-rich elements

**GenBank**	**Symbol**	**Regulation by NO^•^**	**Reported regulation by p38 MAPK**^**a**^
X54150	FCAR	UP	
L06633	PSCDBP	UP	
K03195	SLC2A1	UP	
X59834	GLUL	UP	
U03398	TNFSF9	UP	
M59465	TNFAIP3	UP	UP [77]
U15174	BNIP3	UP	
D86962	GRB10	UP	
M60278	DTR	UP	UP [77]
M69043	NFKBIA	UP	
M92357	TNFAIP2	UP	
U48807	DUSP4	UP	
M16750	PIM1	UP	UP [77]
X89398	UNG	UP	UP [77]
S49592	E2F1	UP	UP [66]
U70426	RGS16	UP	
X02910	TNF	UP	UP [87, 88]
X04500	IL1B	UP	UP [77, 87]
M20681	SLC2A3	UP	
M28130	IL8	UP	UP [77, 89]
M57731	GRO2	UP	UP [77]
U71203	RIT	UP	
S81914	IER3	UP	
J04076	EGR2	UP	
D90070	PMAIP1	UP	UP [77]
J04111	JUN	UP	UP [90]
U09579	CDKN1A	UP	UP [42]
D16532	VLDLR	UP	
U14518	CENPA	DOWN	
U67369	GFI1	DOWN	
U22376	c-Myb	DOWN	
U66838	CCNA1	DOWN	DOWN [91]
M25753	CCNB1	DOWN	DOWN [74]

^a ^As reported in the literature; citations shown in parentheses

### Validation of NO^•^-regulated genes

To determine whether NO^• ^and cAMP effects on mRNA, as measured by microarray, produced downstream effects on secreted protein, TNFα, IL-8 and IL-1β were measured in supernatants collected from parallel cell cultures incubated for 24 h. S-nitrosoglutathione (GSNO) significantly increased TNFα, IL-8, and IL-1β protein [see Additional file 3, part A (*P *< 0.0001 for all)]. In contrast, cAMP decreased TNFα (*P *< 0.0001), increased IL-1β (*P *= 0.003), and had no significant effect on IL-8. NO^•^-induced changes in transcript abundance as determined by microarray were consistent with these results (see Additional file 3, part B].

**Figure 2:**
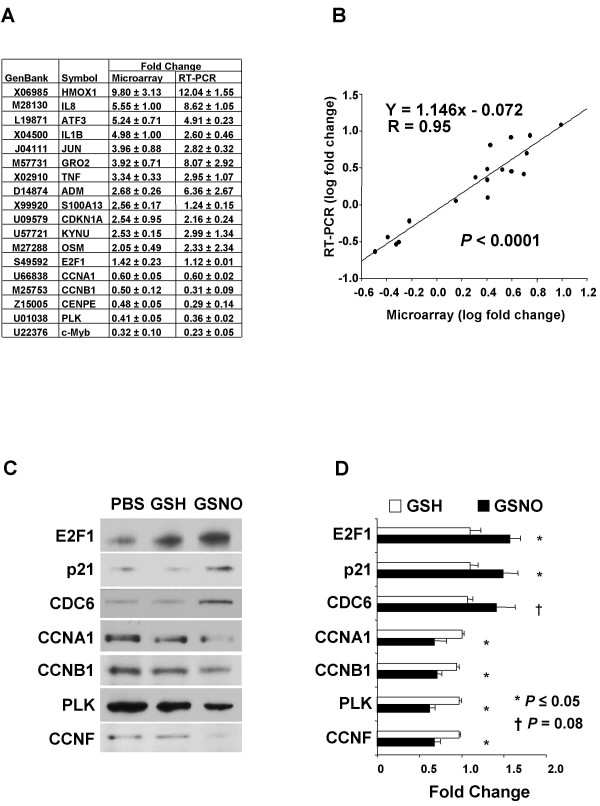
**Validation of microarray results by real-time, reverse transcription (RT) PCR and Western blotting. ***(A) *Fold changes for microarray and RT-PCR at 6 h comparing S-nitrosoglutathione (GSNO; 400 μM) to glutathione (GSH; 400 μM) incubated cells. *(B) *Correlation of fold change comparing the two methods after logarithmic transformation. Results are means ± SE of the last three microarray experiments for which material was available for RT-PCR (performed in triplicate). *(C) *Representative Western blots of cell cycle genes detected by specific antibody and enhanced chemiluminescence. Differentiated U937 cells (1 × 10^7^) were incubated with PBS, GSH (400 μM) or GSNO (400 μM) for 12 h and then lysed for Western blotting. *(D) *Western blot results quantified with laser densitometry and expressed as ratios relative to PBS control values. Data are means ± SE of three or four independent experiments.

Real-time RT- PCR (TaqMan^®^) was used to validate NO^•^-mediated changes in mRNA levels (Fig. 2A and 2B). Of 18 selected genes, 13 were NO^• ^up-regulated, and 5 were down-regulated. Fold changes from microarray experiments strongly correlated with results from RT-PCR (R = 0.95, *P *< 0.0001).

Western blotting of key cell cycle genes regulated by NO^• ^was performed to test whether microarray results accurately predicted changes in protein expression (Fig. 2C and 2D). Three induced genes, E2F transcription factor 1 (E2F1), p21/Waf1/Cip1 (Cdk inhibitor; *CDKN1A*), and cell division cycle 6 *(CDC6) *were examined. E2F1 and p21 are well-characterized master regulatory proteins that control the cell cycle. Four repressed genes, cyclin A1 (*CCNA1*), cyclin B1 (*CCNB1*), polo-like kinase (*PLK*) and cyclin F (*CCNF*) were also measured by Western blotting. In all cases, directional changes in protein expression were consistent with the differential effect of NO^• ^on corresponding transcripts as determined by microarray analysis.

### NO^•^-regulation of cell cycle genes independent of cAMP

NO^• ^induces TNFα by decreasing intracellular cAMP; dibutyryl-cAMP (Bt_2_cAMP), a cell permeable cAMP analog, blocks this effect [9,10]. Moreover, cAMP is an omnipresent second messenger that affects cell proliferation and the cell cycle in a variety of contexts [31-33]. Therefore, Bt_2_cAMP was added to some conditions to test for the cAMP-dependence of NO^•^-mediated effects. Of 106 Bt_2_cAMP-responsive genes, 16 of 103 (16%) compared to 407 of 4870 on the microarray (8%), as annotated in the GO Biological Process database, were cell cycle related, but this effect was not statistically significant (*P *= 0.5). Only one additional cAMP-responsive gene was subsequently annotated to the cell cycle by searching PubMed [see Additional file 4]. However, like NO^•^, cAMP-regulated genes did contain ARE more frequently than expected [[24] of 106 (23%) compared to 540 of 5086 (11%) on the microarray; *P *= 0.0003]. This finding was consistent with the known ability of cAMP to stabilize transcripts that contain ARE [34].

**Figure 3:**
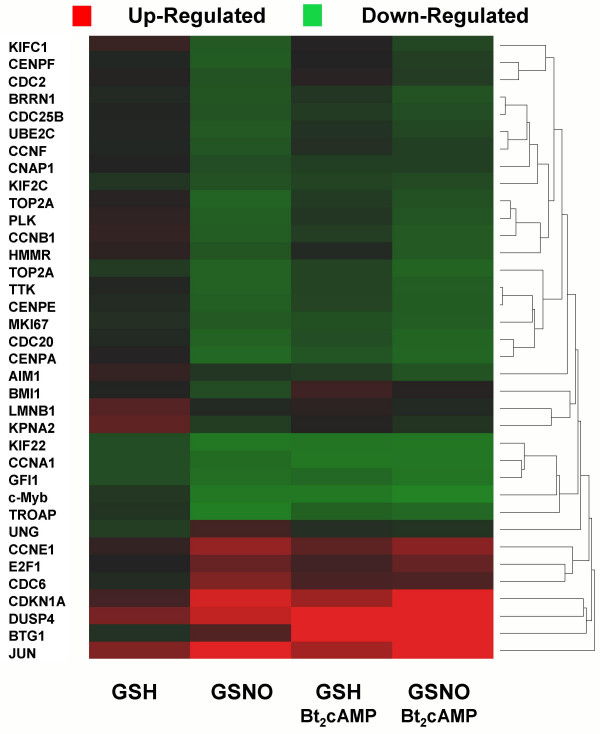
**Heat map of NO^• ^and cAMP effects on the expression of NO^•^-regulated cell cycle genes. **Differentiated U937 cells were incubated with glutathione (GSH; 400 μM) or S-nitrosoglutathione (GSNO; 400 μM) in the absence or presence of dibutyryl-cAMP (Bt_2_cAMP; 100 μM). Color intensity reflects fold change from differentiated cells at 0 h; up-regulation is shown in red and down-regulation in green. Fold changes were computed from the mean results of seven independent microarray experiments.

To further compare the effects of NO^• ^and cAMP, a hierarchical cluster analysis was performed using the 35 cell cycle genes regulated by NO^• ^(Fig. 3). For each of the 5 cell cycle genes significantly affected by both NO^• ^and Bt_2_cAMP [c-Myb, B-cell translocation gene 1 (*BTG1*), dual specificity phosphatase 4 (*DUSP4*), growth factor independent 1 (*GFI1*), and cyclin A1 (CCNA1)], the direction of regulation was the same. Further, for cell cycle genes regulated by NO^•^, cAMP analog either had no effect on or produced expression changes that were similar to and additive with those observed for NO^• ^(Fig. 3). These results suggest that NO^• ^effects on cell cycle genes are independent of its interference with cAMP signaling, since cAMP analog (the opposite signal) was not antagonistic to the actions of NO^•^.

### Analysis of NO^• ^effects on the cell cycle

NO^• ^causes arrest in either the G1 or G2/M phase of the cell cycle [19,35-37]. However, the mechanisms underlying this effect are not well characterized. Annotation of NO^•^-regulated genes to their respective phase of the cell cycle revealed that expression changes were not random (Table 1). Most NO^• ^up-regulated genes (7/8) were G1/S associated, while down-regulated genes were strikingly G2 and G2/M phase associated (24/27). The latter included topoisomerase II alpha (*TOP2A*), cyclin B1, *PLK*, and *CDC25B*, genes that are necessary factors for mitosis. These results show that NO^• ^suppresses the cell cycle in early G2/M by triggering a highly integrated program of gene regulation that does not require soluble guanylate cyclase or cGMP.

**Table 1:** NO^•^-regulated cell cycle related genes

**GenBank**	**Symbol**	**Functional category**	**Fold change**^a^
**G1/S**			
J04111	JUN	Regulation of cell cycle	3.68 ± 0.76
U09579	CDKN1A (p21)	CDK inhibitor	2.06 ± 0.58
U77949	CDC6	DNA replication	1.95 ± 0.48
M74093	CCNE1	Cell cycle control	1.77 ± 0.31
X89398	UNG	DNA repair	1.68 ± 0.30
S49592	E2F1	G1 phase of mitotic cell cycle	1.60 ± 0.19
X61123	BTG1	Negative regulation of cell proliferation	1.60 ± 0.28
L13689	BMI1	Modifies chromatin	0.67 ± 0.09
U67369	GFI1	G1/S-specific transcription in mitotic cell cycle	0.54 ± 0.06
U22376	c-Myb	Cell cycle control	0.30 ± 0.10
**G2**			
U66838	CCNA1	Regulation of CDK activity	0.60 ± 0.12
Z36714	CCNF	Regulation of cell cycle	0.60 ± 0.11
J04088			0.57 ± 0.14
L47276	TOP2A ^b^	Spindle assembly, chromsome condensation	0.42 ± 0.07
X05360	CDC2	Start control point of mitotic cell cycle	0.57 ± 0.11
U28386	KPNA2	Cytoskeleton organization and biogenesis	0.51 ± 0.05
D14678	KIFC1	Spindle assembly, chromsome condensation	0.44 ± 0.10
U14518	CENPA	Chromosome organization and biogenesis	0.38 ± 0.06
**G2/M**			
U48807	DUSP4	Regulation of cell cycle	1.40 ± 0.35
U63743	KIF2C	Microtubule motor activity	0.72 ± 0.10
U83115	AIM1	Tumor supressor, cytoskeleton	0.71 ± 0.07
M34458	LMNB1	Cytoskeletal anchoring	0.66 ± 0.16
D63880	CNAP1	Mitotic surveilance	0.63 ± 0.05
X65550	MKI67	Chromatin/chromosome structure	0.61 ± 0.09
S78187	CDC25B	Cell cycle control	0.60 ± 0.05
D38553	BRRN1	Chromatid separation	0.59 ± 0.08
U73379	UBE2C	Protein degradation	0.54 ± 0.08
U30872	CENPF	Mitosis	0.53 ± 0.07
Z15005	CENPE	Mitotic chromosome movement	0.53 ± 0.10
U29343	HMMR	Mitotic surveilance, cell motility	0.51 ± 0.11
U05340	CDC20	Ubiquitin-dependent protein degradation	0.49 ± 0.07
M86699	TTK	Spindle assembly/mitotic checkpoint	0.47 ± 0.05
U01038	PLK	Mitosis	0.44 ± 0.04
M25753	CCNB1	Mitotic checkpoint	0.43 ± 0.07
D38751	KIF22	Mitosis	0.40 ± 0.18
U04810	TROAP	Cell adhesion	0.24 ± 0.03

^a ^Fold change comparing glutathione (GSH) to S-nitrosoglutathione (GSNO)-treated cells, expressed as the mean ± SE (N = 7)

^b ^Represented by more than one probe set on the microarray that reached statistical significance; each result is shown

**Figure 4:**
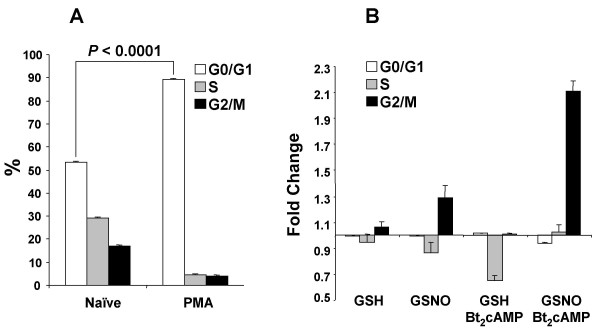
**Cell cycle analysis of U937 cells. ***(A) *U937 cells (1 × 10^6^) were differentiated with PMA (100 nM) for 48 h and then compared with naïve cells for cell cycle distribution using propidium iodide staining. The cell cycle distribution of stained cells was examined by flow cytometry. The percentage of cells in G0/G1, S, and G2/M was determined using ModFit software. *(B) *U937 cells (1 × 10^6^) differentiated as in *A *were treated for 24 h with medium alone, medium with glutathione (GSH; 400 μM) or medium with S-nitrosoglutathione (GSNO; 400 μM) in the absence or presence of dibutyryl-cAMP (Bt_2_cAMP; 100 μM). Cell cycle distribution data, presented as fold change (percentage of cells in each phase of the cell cycle relative to medium alone), are means ± SE of four independent experiments.

To further test this hypothesis, cell cycle analysis was performed on U937 cells using flow cytometry. PMA-differentiation significantly increased the portion of cells in G0/G1 (*P *< 0.0001), while decreasing cells in S (*P *= 0.0007) and G2/M (*P *= 0.003) compared to a naïve, undifferentiated cell population (Fig. 4A). Differentiated cells were then treated with glutathione (GSH) or GSNO in the absence or presence of Bt_2_cAMP. Consistent with NO^•^-induced changes in mRNA expression at 6 h, cell cycle analysis at 24 h demonstrated that NO^• ^increased the portion of cells in G2/M (*P *= 0.0004), and in combination with Bt_2_cAMP, NO^• ^synergistically increased G2/M phase cells (Fig. 4B; *P *= 0.008).

### NO^• ^induction of p21, a master cell cycle regulator; dependence on p38 MAPK and role of mRNA stabilization

The Cdk inhibitor, p21 is known to induce cell cycle arrest in late G1 or early G2/M, [38-40] effects similar to those of NO^•^. NO^• ^increased both p21 mRNA and protein expression in the current experiments. We have previously shown that NO^• ^activates p38 MAPK in U937 and THP-1 cells [13,41]. Activation of p38 MAPK induces p21 in other cell types, [42] and like NO^• ^here, can trigger G2/M cell arrest [40]. We therefore reasoned that NO^• ^might up-regulate p21 by activating p38 MAPK in the present system.

**Figure 5:**
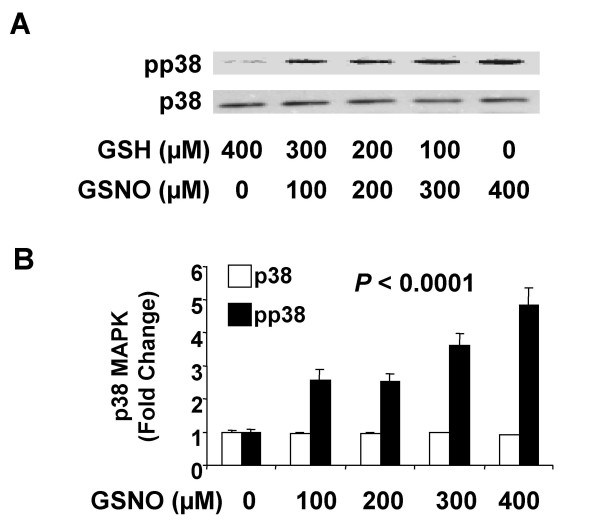
**Effect of NO^• ^on p38 MAPK phosphorylation. **Differentiated U937 cells (5 × 10 ^6^) were incubated with S-nitrosoglutathione (GSNO; 0–400 μM) for 30 min; cells were then lysed for Western blotting to detect total (p38) and phosphorylated forms (pp38) p38 MAPK. *(A) *Representative gel for Western blotting. *(B) *Western blotting results were quantified with laser densitometry and expressed as ratios relative to control values (GSNO = 0 μM). Data are means ± SE of three independent experiments.

**Figure 6:**
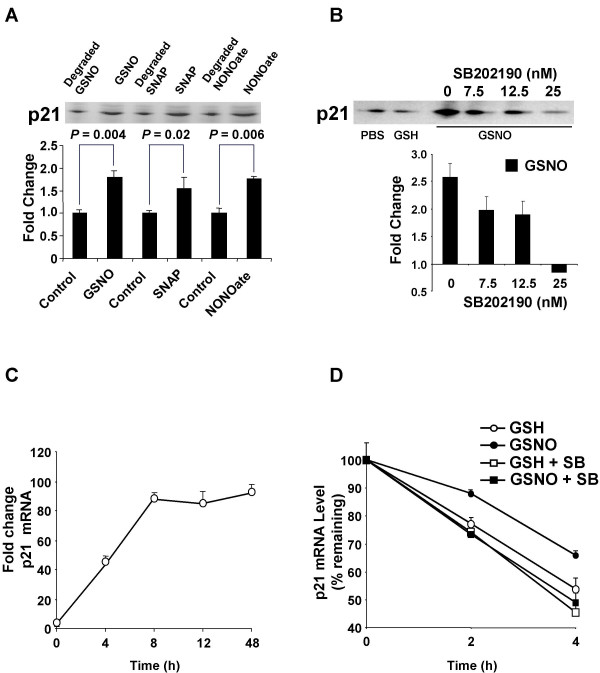
**Effect of NO^• ^donors and p38 MAPK inhibition on p21 expression and mRNA stabilization. ***(A) *Differentiated U937 cells (1 × 10^7^) were incubated with NO^• ^donors, S-nitrosoglutathione (GSNO; 400 μM), S-nitroso-N-acetylpenicillamine (SNAP; 400 μM), or DETA-NONOate (1 mM) or their degraded controls. Western blotting was then performed to detect p21 expression after 12 h of incubation. Results were quantified with laser densitometry and expressed as ratios relative to their appropriate degraded control. Data are means ± SE of four independent experiments. *(B) *Differentiated U937 cells (1 × 10^7^) were incubated with increasing concentrations of the p38 inhibitor SB202190 (0 nM to 25 nM) for 30 min, then exposed to PBS, glutathione (GSH; 400 μM) or GSNO (400 μM) for 12 h. Western blotting was performed to detect p21 expression. Results were quantified with laser densitometry. Data, presented as fold change relative to PBS control values, are means ± SE of three independent experiments. Next, TaqMan^® ^RT-PCR was used to quantify p21 mRNA levels normalized to GAPDH mRNA. *(C) *Changes in p21 mRNA levels during differentiation of U937 cells (1 × 10^7^) with PMA. Data, presented as fold change relative to mean mRNA level in naïve cells, are means ± SE of three independent experiments. *(D) *NO^• ^stabilization of p21 mRNA is dependent on p38 MAPK. U937 cells (1 × 10^7^) were differentiated with PMA for 8 h. After 30 min pretreatment with actinomycin D (2.5 μg/ml) without and with SB202190 (0.1 μM), cells were further incubated with GSH (400 μM) or GSNO (400 μM) for 2 to 4 h. At the specific time points, cells were harvested for total RNA extraction. Data, presented relative to mRNA level at 0 h (arbitrarily set to 100%), are means ± SE of three independent experiments.

GSNO was first confirmed in PMA-differentiated U937 cells to dose-dependently increase p38 MAPK activation. This effect reached significance at the lowest (100 μM) GSNO concentration tested (*P *≤ 0.0001; Fig. 5). Next, three chemically distinct NO^• ^donors were tested for their ability to up-regulate p21 protein in PMA-differentiated U937 cells. GSNO, S-nitroso-N-acetylpenicillamine (SNAP), and DETA-NONOate similarly increased p21 expression in these cells compared to degraded controls (Fig. 6A; *P *< 0.05 for all). Thus, independent of cGMP and type of donor molecule, NO^• ^consistently increased the expression of p21 protein in U937 cells. A specific p38 MAPK inhibitor (SB202190) was used to determine whether blocking this pathway could prevent NO^• ^induction of p21. As shown in Fig. 6B, SB202190 dose-dependently reduced NO^•^-induced p21 protein expression (*P *= 0.0005). Collectively, these results suggest that NO^• ^induces p21 through p38 MAPK activation. Finally, we investigated the effects of NO^• ^and p38 MAPK inhibition on the stability of p21 mRNA, which harbors ARE in its 3' UTR [43]. After 8 h of PMA exposure, p21 expression increased almost 100 fold compared to naïve U937 cells (Fig. 6C). NO^• ^stabilized p21 mRNA in the absence of SB202190 (Fig. 6D;*P *= 0.004), but had no effect in the presence of SB202190 (*P *= 0.5).

### NO^• ^regulation of the cell cycle through E2F1 and p21

The E2F family of transcription factors and p21 act as master regulatory switches that control the cell cycle. E2F1 regulates target genes through E2F-binding sites and thereby plays an essential role in DNA synthesis and the G1/S transition [44-47]. Some p21 effects are mediated by inhibition of E2F factor binding, while other downstream targets contain cell cycle dependent element/cell cycle gene homology region [CDE/CHR] repressor sites within their promoters [27,28,39,48-50]. Protein binding to CDE/CHR sites, triggered by p21 expression, causes repression of a diverse group of cell cycle genes and subsequent late G1 or early G2/M phase arrest, responses that are highly similar to NO^• ^effects shown here. The ability of NO^• ^to increase the expression of E2F1 and p21 may explain much of its broad control over the cell cycle that ultimately involves dozens of gene products. We therefore identified NO^•^-regulated genes that contain E2F or CDE/CHR promoter sites by searching **TRANSFAC **[51] and PubMed[52]. Sixteen NO^•^-regulated genes contain apparent E2F sites (Table 3A). Seven of these are annotated to the G1/S phase of the cell cycle, six of which have reported E2F1 responses that are concordant with NO^• ^effects in the current experiment. Notably, of 10 NO^• ^down-regulated transcripts with possible E2F-binding sites, only c-Myb is a G1/S phase gene. Further, 5 of these genes are known to also contain a CDE/CHR binding site that appears to be functionally dominant (Table 3B). Moreover, of the 27 cell cycle genes down-regulated by NO^•^, 8 are known targets of p21 repression (all G2/M associated), including 6 genes with putative CDE/CHR sites (Table 3B) and two others, lamin B1 (LMNB1) and centromere protein F (CENPF) [39].

**Table 3A:** Specific promoter elements associated with NO^•^-regulated cell cycle genes

**3A. Genes with E2F sites**				
**GenBank**	**Symbol**	**Cell cycle phase**	**Regulation by NO^•^**	**Regulation by E2F1**^**a**^
J04111	JUN	G1/S	Up	Up [63]
U09579	CDKN1A	G1/S	Up	Up [59]
U77949	CDC6	G1/S	Up	Up [61]
M74093	CCNE1	G1/S	Up	Up [63]
X89398	UNG	G1/S	Up	Up [64]
S49592	E2F1	G1/S	Up	Up [61]
U22376	c-Myb	G1/S	Down	Up [63, 92]
U66838	CCNA1 ^b^	G2	Down	Up [93]
J04088	TOP2A^b^	G2	Down	Up [62]
L47276	TOP2A ^b^	G2	Down	Up [62]
X05360	CDC2 ^b^	G2	Down	Up [62, 64]
X65550	MKI67	G2M	Down	Up [63]
U14518	CENPA ^b^	G2/M	Down	
S78187	CDC25B	G2/M	Down	
M86699	TTK	G2/M	Down	
U01038	PLK ^b^	G2/M	Down	
M25753	CENPE	G2/M	Down	

^a ^As reported in the literature; citations shown in parentheses

^b ^These genes also contain CDE/CHR sites as shown in Table 3B

**Table 3B:** Specific promoter elements associated with NO^•^-regulated cell cycle genes

**3B. Genes with CDE/CHR sites**				
**GenBank**	**Symbol**	**Cell cycle phase**	**Regulation by NO^•^**	**Reported regulation by p21**^**a**^
U66838	CCNA1 ^b^	G2	Down	Down
J04088	TOP2A ^b^	G2	Down	Down
L47276	TOP2A ^b^	G2	Down	Down
X05360	CDC2 ^b^	G2	Down	Down
U14518	CENPA ^b^	G2/M	Down	Down
U01038	PLK ^b^	G2/M	Down	Down
M25753	CCNB1	G2/M	Down	Down

^a ^As reported in the literature [39]

^b ^These genes also contain E2F sites as shown in Table 3A

**Figure 7:**
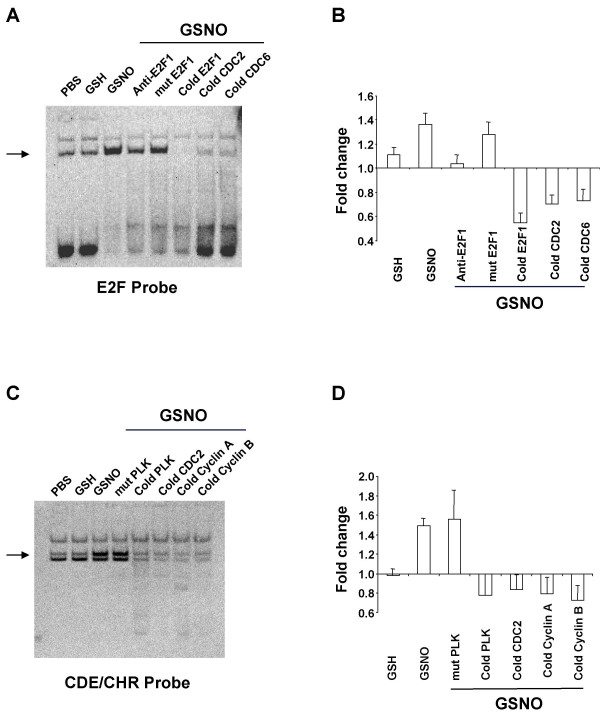
**NO^• ^increases protein binding to E2F and CDE/CHR promoter sites. ***(A) *Representative gel for protein binding to the E2F probe detected by ECL. *(B) *Results for the E2F probe quantified with laser densitometry. Data, presented as ratios relative to PBS values (set to 1), are means ± SE of six independent experiments. *(C) *Representative gel for protein binding to CDE/CHR probe detected by ECL. *(D) *Results for the CDE/CHR probe quantified with laser densitometry. Data, presented as ratios relative to PBS values (set to 1), are means ± SE of six independent experiments. Differentiated U937 cells were incubated with glutathione (GSH; 400 μM) or S-nitrosoglutathione (GSNO; 400 μM) for 3 h. Nuclear extract (15 μg) was prepared and incubated with double-stranded, biotin-N4-CTP labeled DNA probe representing the E2F or CDE/CHR concensus sequence from the E2F1 or *PLK *promoter, respectively. Protein binding was then determined by electrophoretic mobility shift assay. Complexes were competed with 100-fold molar excess of cold probes, site-directed mutagenesis of the consensus sequence, and for the E2F probe, *E2F1*-directed antibody as indicated to test for binding specificity.

Next, electrophoretic mobility shift assays (EMSA) were performed to test whether NO^• ^altered protein binding to E2F and CDE/CHR consensus sequences. PMA-differentiated U937 cells were treated with PBS, GSH, or GSNO followed by preparation of nuclear extract. NO^• ^increased binding to both E2F (Fig. 7A and 7B) and CDE/CHR probes (Fig. 7C and 7D). Site-directed mutagenesis of each consensus sequence abolished competition (Fig. 7) and E2F1-directed antibody blocked complex formation with labeled E2F probe (Fig. 7A and 7B).

### Summary

**Figure 8:**
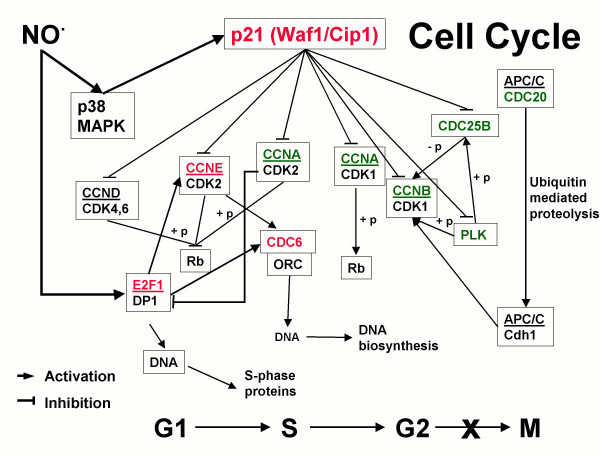
**Schematic representation of NO^• ^regulation of the cell cycle. **Genes differentially regulated in this investigation are shown in color; red signifies up- and green down-regulation. At the bottom, NO^• ^is depicted as allowing progression to G2 where it induces cell cycle arrest. KEGG pathway [86] reproduced and Modified with permission.

NO^•^, independent of cGMP, regulated a diverse subset of genes involved in inflammation, metabolism, apoptosis, the cell cycle, proliferation, signal transduction, and transport. Notably, genes associated with the cell cycle and proliferation, including the master cell cycle regulatory genes E2F1 and p21, were over-represented. Further, NO^•^-regulated transcripts had ARE (post-transcriptional regulatory sites) in their 3' UTR and were p38 MAPK responsive more frequently than expected. E2F1 induction by NO^• ^was associated with up-regulation of several genes involved in G1/S transition that contain E2F-binding sites. NO^• ^also induced p21, an ARE-containing gene, through p38 MAPK activation and mRNA stabilization. This was associated with the down-regulation of G2/M phase genes, at least in part, through changes in protein binding to CDE/CHR promoter sites. Collectively, these results demonstrate that NO^•^, independent of cGMP and cAMP, triggers a specific and highly coordinated genetic program that alters the G1/S transition and induces arrest in early G2/M (Fig. 8). MAPK pathways and mRNA stability are major mechanisms by which NO^• ^regulates the transcriptome.

## Discussion

NO^• ^has potent anti-tumor and anti-atherosclerotic effects that are closely associated with its ability to block cell proliferation [18,53]. This activity of NO^• ^has been ascribed to both cGMP-dependent and -independent mechanisms. Experiments in rodents have found, with a few notable exceptions [54,55], that NO^• ^controls the cell cycle through cGMP. These studies have focused on the importance of a NO^•^-cGMP-PKA-ERK 1/2 signal transduction pathway [22-24]. Accordingly, cAMP itself has also been reported to inhibit cell proliferation through activation of PKA and/or ERK 1/2 with the up-regulation of p27 or p21 in a cell-specific manner [31-33,56]. In contrast, the anti-proliferation effects of NO^• ^in human cells have been frequently associated with cGMP-independent signaling [19,29]. Here, a transcriptome-wide approach revealed that NO^• ^exerts broad control over the cell cycle through p38 MAPK activation and mRNA stabilization.

In a previous study, we found that NO^• ^up-regulates TNFα by decreasing cAMP, an effect antagonized by cAMP analogs. Therefore Bt_2_cAMP was used in this investigation to explore whether some effects of NO^• ^on the transcriptome could be attributed to its interaction with cAMP signaling. However, our results indicate that NO^•^-cAMP signaling appears to be a minor pathway, regulating less than 6 of the affected transcripts in U937 cells (data not shown). These included TNFα, as well as pim-1 oncogene *(PIM1)*, TNFα-induced protein 2 (*TNFAIP2*), and glutathione reductase *(GSR)*. Importantly, for cell cycle genes, NO^• ^and Bt_2_cAMP consistently had the same directional effect on transcripts, although NO^• ^regulated more genes overall. Thus, decreases in intracellular cAMP appear unrelated to NO^• ^effects on the cell cycle. Furthermore, treatment with both NO^• ^and Bt_2_cAMP synergistically provoked cell cycle arrest in G2/M, suggesting that NO^•^-induced decreases in cAMP may attenuate some of its effects on the cell cycle. Although this experiment also provides useful intormation on gene regulation by cAMP in U937 cells, the reader should keep in mind that Bt_2_cAMP was the only analog studied and some effects may have been caused by its butyryl component.

U937 cells were PMA-differentiated in the current experiments to render them capable of producing TNFα and IL-8, two cytokines previously identified as NO^•^-responsive [7-13]. Further, this treatment also reduced cell proliferation and forced >80% of the cells into the G0/G1 phase of the cell cycle, allowing for a more coherent analysis of cell cycle regulation (Fig. 4). However, PMA itself had large effects on NO^•^-regulated genes such as p21 (Fig. 6D) and the findings here cannot be extrapolated directly to naïve U937 cells. Fortunately, Turpev and colleagues have recently reported selected microarray results from NO^• ^exposure of undifferentiated U937 and Mono Mac 6 cells [57]. Of interest, a number of key genes identified by this group were also found to be similarly regulated by NO^• ^in PMA-differentiated cells including HMOX1, IL-8, activating transcription factor 4 (ATF4), BCL2/Adenovirus E1B 19 kD-interacting protein 3 (BNIP3), and importantly p21/Waf1/Cip1.

The NO^• ^donor GSNO was found to activate p38 MAPK in U937 cells, which was consistent with our previous result using SNAP, another NO^• ^donor [41]. Furthermore, three different NO^• ^donors were shown here to consistently increase p21 protein expression, indicating that this effect is NO^•^-specific and donor independent. Importantly, very low concentrations of the p38 MAPK inhibitor SB202190 were found to block the induction of p21 protein by NO^•^, further establishing the role of p38 MAPK as an intermediary signal transduction event. Finally, p21 mRNA was measured serially by RT-PCR after transcriptional blockade in the absence or presence of SB202190 showing that this transcript is stabilized by NO^• ^through a p38 MAPK-dependent mechanism.

Others have found that NO^• ^increases p21 mRNA and protein expression in human vascular smooth muscle independent of cGMP [58]. In addition, p38 MAPK activation has been shown to increase p21 expression by both transcriptional activation and protein stabilization [42]. E2F1 is also known to induce p21 transcription [59] providing another mechanism by which NO^• ^may have increased p21 expression in the current experiment. Conversely, as already discussed, NO^• ^decreases cAMP, reducing the ability of Sp1 to bind to GC box elements and thereby repressing the transcription of Sp1-dependent genes such as eNOS [11]. Interestingly, p21 is highly dependent on Sp1 for transcription [60] and is induced by cAMP [56], findings consistent with the possibility that p21 may be transcriptionally repressed by NO^•^-cAMP-Sp1 signal transduction. Nonetheless, NO^• ^induction of p21 demonstrates that other mechanisms dominate over any negative effects of NO^• ^on Sp1 binding to the p21 promoter. Here, we focused on mRNA stabilization, because of the strong indication in our microarray data that this is a major mechanism of gene regulation by NO^•^. However, other mechanisms such as changes in transcription, translation, or protein stability may have contributed substantially to the net effects of NO^• ^on p21 expression.

The E2F family of transcription factors play important roles in G1/S phase transition. E2F1 up-regulates many G1/S phase genes including itself, cyclin E1 (*CCNE1*), *CDC6*, uracil-DNA glycosylase *(UNG)*, JUN, p21 and c-Myb [46,47,61-65]. Except for c-Myb, all were up-regulated by NO^• ^in the present study, suggesting that NO^• ^may drive differentiated U937 cells through the G1/S transition by inducing E2F1 expression. This conclusion is further supported by EMSA showing that NO^• ^increases E2F1 binding to E2F consensus sequence. However, increased E2F1 expression may not be the only mechanism contributing to these observed changes in DNA binding. NO^• ^activates p38 MAPK in U937 cells and p38 MAPK has been shown to increase E2F1 binding to E2F sites [66]. Further, cyclin A1 was down-regulated by NO^• ^and has been shown to turn off E2F1 target genes by decreasing E2F1 DNA binding [47,67].

Notably, c-Myb and a number of G2 or G2/M phase genes that contain E2F sites were down-regulated by NO^•^. E2F sites can function as repressors in some genes and their disruption by mutation leads to promoter activation [46,61,68]. Further, NO^•^-responsive genes with both E2F elements and CDE/CHR repressor sites were uniformly down-regulated. Promoters with CDE/CHR motifs are repressed by p21 [28,39], which was also induced by NO^•^. Therefore, even for promoters activated by E2F1, repression through CDE/CHR sites appears to be the dominant action of NO^• ^in this cellular context. Moreover, E2F and CHR sites may cooperate as co-repressors [69]. Although *CDC25B *lacks an identifiable CDE/CHR site, it does have a proximal repressor and its regulation is similar to CDE/CHR-containing genes [70].

Cell cycle arrest induced by p21 occurs in late G1/S [40,71] or early G2/M [38] and is mediated, at least in part, by the repression of target genes with CDE/CHR sites [28]. CDE/CHR sites are present in the promoters of cyclin A1 [50], *CDC2 *[48], cyclin B1 [72], and *TOP2A *[49], centromere protein A (*CENPA*) [73] and *PLK *[27]. All of these genes are G2 or G2/M related and are down-regulated by p21, results consistent with the effects of NO^• ^observed here. EMSA confirmed the hypothesis that NO^• ^regulates protein binding to CDE/CHR sites. Collectively, these findings suggest that NO^• ^regulates many G2/M phase cell cycle genes through p21. However, NO^• ^may also regulate some of these downstream p21 targets through additional mechanisms. For example, c-Myb, cyclin A1, cyclin B1, and *CENPA *have ARE in their 3' UTR, indicating that NO^• ^might alter the stability of these transcripts. Notably, cyclin A1 and cyclin B1 are down-regulated by p38 MAPK, a signal transduction pathway that was activated by NO^• ^in the current experiments. Importantly, p38 MAPK has been shown to induce cell cycle arrest at the G2 checkpoint through mechanisms that were only partially dependent on p21 [74].

ARE in 3' UTR have been implicated in the control of transcript stability and have an important post-transcriptional impact on transcriptome content [11,75-77]. We previously demonstrated that independent of cGMP, NO^• ^up-regulates IL-8, but not TNFα post-transcriptionally through p38 MAPK activation [13]. In the current investigation, ARE- containing genes including IL-8 and p21 were over-represented among NO^•^-regulated genes. Nearly half of these ARE genes have been reported to be regulated by p38 MAPK (Table 2). Notably, NO^• ^responses were all in the same direction as those reported for p38 MAPK activation. Previous microarray experiments that globally tested mRNA stability found that 10% of transcripts were associated with p38 MAPK-dependent regulation [77]. The over-representation of p38 MAPK-regulated genes in our experiments indicates that this stress kinase is an important target of NO^•^.

## Conclusion

The present investigation was focused on understanding cGMP-independent gene regulation by NO^•^. Major themes within the identified gene list were the predominance of cell cycle-related genes and ARE-containing transcripts. NO^• ^was found to trigger a specific and coordinated cell cycle arrest independent of both cGMP and cAMP. E2F1 induction up-regulated target genes involved in G1/S transition through E2F sites. NO^• ^stabilization of p21 mRNA was p38 MAPK dependent and led to increased protein binding to CDE/CHR promoter sites and the down-regulation of G2/M phase genes. The cell cycle is a major target of NO^•^-mediated gene regulation. Importantly, p38 MAPK and mRNA stability are major intermediary mechanisms through which NO^• ^affects the human transcriptome.

## Methods

### Reagents and cell culture

PMA, GSNO, S-nitroso-N-SNAP, Bt_2_cAMP and SB202190 were purchased from Calbiochem (San Diego, CA). DETA-NONOate was obtained from Cayman (Ann Arbor, Michigan); actinomycin D and GSH were from Sigma-Aldrich (St. Louis, MO). U937 cells (ATCC, Rockville, MD), a human monoblastoid line devoid of NO^• ^sensitive guanylate cyclase, [9,30] were cultivated and then differentiated with PMA (100 nM) for 48 h as described previously [9].

### Microarray experiments

NO^• ^donor, GSNO (400 μM), or its precursor GSH (400 μM) was added into differentiated U937 cells in the absence or presence of Bt_2_cAMP (100 μM) followed by incubation at 37°C for 6 h (N = 7). Cells were then washed three times with ice cold PBS. Total RNA was extracted using RNeasy Mini kits (Qiagen, Valencia, CA) and reverse transcribed (10 μg) using the SuperScript II^® ^custom kit (Invitrogen, Carlsbad, CA). Resulting cDNA (1 μg) was *in vitro *transcribed into biotin-labeled cRNA using the BioArray high yield RNA transcript labeling kit (Enzo Life Sciences, Farmingdale, NY). After fragmentation, biotin-labeled cRNA (20 μg) was hybridized to Affymetrix HuGeneFL 6800^® ^microarrays [> 5,000 unique transcripts after masking uninformative probe sets [Affymetrix Website, #106] following the Affymetrix protocol [78]. After staining with streptavidin phycoerythrin (Molecular Probes) and enhancing with anti-streptavidin (0.5 mg/ml, Vector Laboratories, Burlingame, CA), microarrays were scanned using Agilent GeneArray Scanner.

### Cytokine assay and TaqMan^® ^Real time RT-PCR

Supernatants were collected from duplicate cell cultures after 24 h of incubation. TNFα, IL-8 and IL-1β production were measured using ELISA kits (R & D Systems, Minneapolis, MN) according to the manufacturer's instructions.

The TaqMan^® ^Real time RT-PCR system (Applied Biosystems, ABI, Rockville, MD) was employed to quantify mRNA levels. Gene-specific TaqMan^®^probes and PCR primers were designed using Primer Express 1.0 (ABI) and their sequences are provided in supplemental data [see Additional file 5]. The High-capacity cDNA Archive kit (ABI, Foster City, CA) was employed to prepare cDNA from 2 μg of total RNA. The resulting cDNA was used for RT-PCR in triplicate according to the standard ABI protocol. The average quantities of target gene mRNA relative to GAPDH mRNA was determined for each sample. The target gene/GAPDH ratio in GSH treated cells was arbitrarily set at 1 and results from all other samples were expressed relative to that standard.

### Western blotting

Polyclonal antibodies detecting p21, cyclin A1, cyclin B1, *CDC6*, E2F1 and cyclin F were obtained from Santa Cruz Biotechnology (Santa Cruz, CA). *PLK *antibody was purchased from BD Transduction Laboratories (San Diego, CA). Aliquots of 1 × 10^7 ^cells were incubated with PBS, GSH (400 μM), or GSNO (400 μM) for 12 h to prepare whole cell lysates. Separate experiments were conducted to detect total and phosphorylated p38 MAPK using anti-p38 and anti-pp38 (Promega, WI). All Western blotting was performed using 20 μg of whole cell lysate as previously described [13].

### Cell cycle analysis

Cells were harvested and stained with propidium iodide and the cell cycle distribution of stained cells was determined by flow cytometry (FACS Calibur, Becton Dickinson). The percentage of cells in G0/G1, S, and G2/M was determined using ModFit (Verify Software House Inc., Topsham, ME) and expressed as relative change compared to PMA-differentiation alone. Naïve U937 cells were compared to cells (1 × 10^6^) incubated with PMA for 48 h to examine the effects of differentiation on the cell cycle. As expected, PMA differentiation pushed cells into G0/G1 arrest (>80% of cells). These cells were then treated with GSH (400 μM) or GSNO (400 μM) without or with Bt_2_cAMP for 24 h and processed for cell cycle analysis as described above.

### EMSA

Differentiated U937 cells were cultured for 3 h with GSH (400 μM) or GSNO (400 μM). EMSA were performed with 15 μg of nuclear extract and double-stranded DNA probes labeled with biotin-N4-CTP according to manufacturer's instructions (Pierce, Rockford, Illinois). Probes purchased from Sigma-Genosys (The Woodlands, TX) were as follows: E2F probe (5'-CGTGGCTCTTTCGCGGCAAAAAGGA-3') representing the -39 to -15 section of the E2F1 promoter and CDE/CHR probe (5'-GTTCCCAGCGCCGCGTTTGAATTC-3') representing the -10 to +14 section of human *PLK *promoter. Binding complexes were competed using 100-fold molar excess of cold probes. Sequences for these cold probes are available in [see Additional file 6].

### Microarray data analysis, gene annotation, and statistics

Images were analyzed using Microarray Suite 4.0 (Affymetrix). Global scaling was set at 100. Data were transformed and analyzed using the MSCL Analyst's Toolbox  written in the JMP scripting language (SAS Institute, Cary, NC). Average difference (AD) values were standardized and transformed using the Symmetric Adaptive Transform [79-81] yielding quantile-normalized, homogenous variance scaled results. Differentially regulated genes were identified from 7 independent experiments using a combination of consistency tests set at a 4% false discovery rate (FDR) and an average AD above 20 for at least one condition. One of 7 experiments was an outlier for some genes, but was not allowed to eliminate genes found significant in the other six. Fold change in gene expression was calculated directly from AD results after raising negative values to 10, and likewise adding 10 to all positive values.

Genes were annotated by searching **NIH-DAVID **[82,83] and PubMed [52]. Over-representation of gene categories among differentially expressed transcripts was tested using **Expression Analysis Systematic Explorer **[84,85]. EASE scores (penalized Fisher exact test), corrected for multiple comparisons using bootstrap resampling with 10,000 iterations, are reported as P-values. These analyses and tests of significance relied on databases within EASE and therefore did not include additional genes that were annotated to particular functional categories using PubMed.

All data not derived from microarrays are presented as mean ± standard error (SE) of at least three independent experiments. All *P*-values are two-sided unless noted otherwise, and considered significant if less than 0.05. To compare treatment effects on cytokine secretion, a two-way ANOVA with blocking for experiment was carried out on the logarithm of the measured concentrations for TNFα, IL-8 and IL-1β (supplemental Fig. 1A). A linear regression of RT-PCR log fold change *versus *microarray log fold change was generated to evaluate the validity of the microarray data (Fig. 2B). To determine whether NO^• ^affected the protein expression of various cell cycle genes, paired t-tests, unadjusted for multiple comparisons, were performed for GSH versus GSNO, after log normalization to PBS (Fig. 2D). Log percentages of naïve and PMA-differentiated U937 cells in each phase of the cell cycle were compared by paired t-tests, unadjusted for multiple comparisons (Fig. 4A). Two-way ANOVAs with blocking were performed on log percentage of cells in each phase of the cell cycle to assess the significance of the NO^• ^effect, cAMP effect, and their interaction (Fig. 4B). Effects of NO^• ^on p38 MAPK phosphorylation (Fig. 5) were investigated with a one-way ANOVA comparing the log fold change of laser densitometry intensity (pp38/p38) over different concentrations of GSNO. A post-hoc Dunnett's test was carried out to determine the lowest concentration at which the effect became significant compared to control. The expression of p21 in the presence of GSNO, SNAP, or DETA-NONOate was compared to that in the presence of their respective degraded controls with paired t-tests, unadjusted for multiple comparisons (Fig. 6A). The dose effect of SB202190 on NO^•^-induced p21 protein expression normalized to PBS was analyzed using a one-way ANOVA (Fig. 6B). NO^• ^stabilization of p21 mRNA over time (with and without SB202190) was assessed using constrained one-way analysis of covariance, after natural log transformation of relative mRNA amounts (Fig. 6D).

## List of abbreviations

ARE: AU-rich elements; Bt_2_cAMP: dibutyryl-cAMP; CDE/CHR: cell cycle dependent element/cell cycle gene homology region; EMSA: electrophoretic mobility shift assays; GO: Gene Ontology; GSH: glutathione; GSNO: S-nitrosoglutathione; NO^•^: nitric oxide; SNAP: S-nitroso-N-acetylpenicillamine; UTR: untranslated regions.

## Authors' contributions

**Xiaolin Cui, Shuibang Wang, and Robert L. Danner**: framing the question and designing experiments; developing downstream hypotheses; writing the manuscript. **Jianhua Zhang**: developing, designing, and performing the real time RT-PCR and EMSA experiments; raising questions that contributed to the scientific process. **Xiaolin Cui and Penglin Ma**: Designing and performing experiments to test downstream hypotheses. Performing Western blotting. Final annotation of gene lists. **Daniela E. Myers, Ilana G. Goldberg and Ana del Pilar Cintron**: developing methodology and performing bench work for microarray experiments including preparation of total RNA, reverse transcription, labeling, hybridization, and scanning. Preliminary annotation of gene lists. **Kelly J. Sittler, Peter J. Munson and Jennifer J. Barb**: developing computation tools and data normalization methods; detecting differentially regulated genes and performing statistical analysis. **J. Philip McCoy**: developing, designing, and performing the cell cycle analysis experiments. Editing the manuscript.

## Supplementary Material

Additional File 1 Classification of NO^•^-Upregulated Genes. Complete list of genes upregulated by NO^• ^Genes are classified by function and fold change from control is shown. Click here for file

Additional File 2 Classification of NO^•^-Downregulated Genes. Complete list of genes downregulated by NO^• ^Genes are classified by function and fold change from control is shown. Click here for file

Additional File 3 Confirmation of NO^• ^effects on TNFα, IL-8 and IL-1β (A) NO^• ^up-regulated secreted TNFα, IL-8 and IL-1β protein at 24 h as measured by ELISA. Dibutyryl cAMP (Bt_2_cAMP) decreased TNFα, increased IL-1β, and had no effect on IL-8. Data are means ± SE of six independent experiments. *(B) *NO^• ^effect on TNFα, IL-8 and IL-1β mRNA at 6 h as measured by microarray (N = 7) were similar to changes in secreted protein. Click here for file

Additional File 4 Classification of cAMP-Regulated Genes. Complete list of genes regulated by cAMP. Genes are classified by function and fold change from control is shown. Click here for file

Additional File 5 RT-PCR Primers and Probes. List of genes tested by RT-PCR including the sequence of primers and probes used in the assays. Click here for file

Additional File 6 Electrophoretic Mobility Shift Assay (EMSA) Probes. List of genes from which E2F and CDE/CHR promoter sequences were derived for testing by electrophoretic mobility shift assay (EMSA). For each gene, the EMSA probe sequence is shown. Click here for file
