# Supplementary material for: cGMP-independent nitric oxide signaling and regulation of the cell cycle
Source: BMC Genomics. 2005 Nov 3;6:151. doi: 10.1186/1471-2164-6-151 (PMC1312313; doi:10.1186/1471-2164-6-151)
Supplement: Additional File 1 — Classification of NO•-Upregulated Genes. Complete list of genes upregulated by NO• Genes are classified by function and fold change from control is shown. [file 1471-2164-6-151-S1.doc]

| Classification of NO·-Upregulated Genes | | | | | |
| --- | --- | --- | --- | --- | --- |
| **GenBank** | **Unigene** | **Name** | | **Symbol** | **Fold change** **^a^** |
| **Metabolism (12)** | | | | | |
| X06985 | Hs.202833 | heme oxygenase (decycling) 1 | HMOX1 | | 9.79±2.53 |
| L35546 | Hs.315562 | glutamate-cysteine ligase, modifier subunit | GCLM | | 4.06±1.02 |
| J03934 | Hs.406515 | NAD(P)H dehydrogenase, quinone 1 | NQO1 | | 2.86±0.52 |
| X97324 | Hs.3416 | Adipose differentiation-related protein | ADFP | | 2.38±0.37 |
| U57721 | Hs.444471 | kynureninase (L-kynurenine hydrolase) | KYNU | | 2.23±0.38 |
| S52028 | Hs.19904 | cystathionase (cystathionine gamma-lyase) | CTH | | 1.99±0.29 |
| U30255 | Hs.392837 | phosphogluconate dehydrogenase | PGD | | 1.97±0.24 |
| X15722 | Hs.414334 | glutathione reductase | GSR | | 1.93±0.53 |
| BC007257 | Hs.533013 | cystathionine-beta-synthase | CBS | | 1.79±0.36 |
| X90858 | Hs.314828 | uridine phosphorylase | UP | | 1.60±0.22 |
| X59834 | Hs.442669 | glutamate-ammonia ligase (glutamine synthase) | GLUL | | 1.54±0.10 |
| L09229 | Hs.406678 | fatty-acid-Coenzyme A ligase, long-chain 1 | FACL1 | | 1.33±0.24 |
| **Inflammation (11)** | | | | | |
| M28130 | Hs.624 | interleukin 8 | IL8 | | 4.25±0.75 |
| M57731 | Hs.75765 | GRO2 oncogene | GRO2 | | 4.25±0.66 |
| X04500 | Hs.126256 | interleukin 1, beta | IL1B | | 3.83±0.80 |
| X02910 | Hs.241570 | tumor necrosis factor (TNF superfamily, member 2) | TNF | | 2.94±0.33 |
| D14874 | Hs.441047 | Adrenomedullin | ADM | | 2.85±0.34 |
| AB000584 | Hs.296638 | Prostate differentiation factor | PLAB | | 2.21±0.41 |
| M92357 | Hs.101382 | tumor necrosis factor, alpha-induced protein 2 | TNFAIP2 | | 1.84±0.24 |
| M59465 | Hs.211600 | tumor necrosis factor, alpha-induced protein 3 | TNFAIP3 | | 1.79±0.21 |
| M69043 | Hs.81328 | nuclear factor of kappa light polypeptide gene enhancer in B-cells inhibitor, alpha | NFKBIA | | 1.75±0.39 |
| M24283 | Hs.386467 | intercellular Adhesion molecule 1 (CD54), human rhinovirus receptor | ICAM1 | | 1.52±0.17 |
| X54150 | Hs.193122 | Fc fragment of IgA, receptor for the Fc region of IgA | FCAR | | 1.46±0.24 |
| **Cell proliferation (9)** | | | | | |
| D90070 | Hs.96 | phorbol-12-myristate-13-acetate-induced protein 1 | PMAIP1 | | 2.94±0.45 |
| D87953 | Hs.318567 | N-myc downstream regulated | NDRG1 | | 2.74±0.48 |
| J04076 | Hs.1395 | early growth response 2 (Krox-20 (Drosophila) homolog) | EGR2 | | 2.61±0.65 |
| M27288 | Hs.248156 | oncostatin M | OSM | | 2.39±0.52 |
| U03398 | Hs.1524 | tumor necrosis factor (ligand) superfamily, member 9 | TNFSF9 | | 1.74±0.21 |
| M60278 | Hs.799 | diphtheria toxin receptor (heparin-binding epidermal growth factor-like growth factor) | DTR | | 1.74±0.28 |
| D86962 | Hs.512118 | growth factor receptor-bound protein 10 | GRB10 | | 1.49±0.19 |
| M16750 | Hs.81170 | pim-1 oncogene | PIM1 | | 1.48±0.13 |
| U46751 | Hs.182248 | sequestosome 1 | SQSTM1 | | 1.40±0.11 |
| **Transcription factors (8)** | | | | | |
| L19871 | Hs.460 | activating transcription factor 3 | ATF3 | | 4.63±0.65 |
| X71427 | Hs.355867 | fusion, involved in t(12;16) malignant liposarcoma | FUS | | 2.63±0.90 |
| J04102 | Hs.292477 | v-ets avian erythroblastosis virus E26 oncogene homolog 2 | ETS2 | | 2.40±0.44 |
| L06633 | Hs.270 | pleckstrin homology, Sec7 and coiled/coil domains, binding protein | PSCDBP | | 2.04±0.31 |
| D90209 | Hs.181243 | activating transcription factor 4 (tax-responsive enhancer element B67) | ATF4 | | 1.67±0.31 |
| X52560 | Hs.99029 | CCAAT/enhancer binding protein (C/EBP), beta | CEBPB | | 1.52±0.32 |
| M62831 | Hs.737 | immediate early protein | ETR101 | | 1.45±0.16 |
| U91616 | Hs.458276 | nuclear factor of kappa light polypeptide gene enhancer in B-cells inhibitor, epsilon | NFKBIE | | 1.45±0.22 |
| **Signal transduction (8)** | | | | | |
| M90657 | Hs.351316 | transmembrane 4 superfamily member 1 | TM4SF1 | | 13.56±4.04 |
| U70426 | Hs.413297 | regulator of G-protein signalling 16 | RGS16 | | 3.71±0.49 |
| U15932 | Hs.2128 | dual specificity phosphatase 5 | DUSP5 | | 3.21±0.45 |
| U71203 | Hs.446472 | Ric (Drosophila)-like, expressed in many tissues | RIT | | 2.40±0.21 |
| X99920 | Hs.446592 | S100 calcium-binding protein A13 | S100A13 | | 2.38±0.32 |
| X57579 | Hs.28792 | inhibin, beta A (activin A, activin AB alpha polypeptide) | INHBA | | 2.17±0.50 |
| S59049 | Hs.75256 | regulator of G-protein signalling 1 | RGS1 | | 1.78±0.21 |
| M62994 | Hs.81008 | filamin B, beta (actin-binding protein-278) | FLNB | | 1.61±0.13 |
| **Cell cycle (8)** | | | | | |
| J04111 | Hs.78465 | v-jun avian sarcoma virus 17 oncogene homolog | JUN | | 3.68±0.76 |
| U09579 | Hs.370771 | cyclin-dependent kinase inhibitor 1A (p21, Cip1) | CDKN1A | | 2.06±0.58 |
| U77949 | Hs.405958 | CDC6 (cell division cycle 6, S. cerevisiae) homolog | CDC6 | | 1.95±0.48 |
| M74093 | Hs.244723 | Human cyclin mRNA | CCNE1 | | 1.77±0.31 |
| X89398 | Hs.78853 | human uracil-DNA glycosylase | UNG | | 1.68±0.30 |
| S49592 | Hs.96055 | E2F transcription factor 1 | E2F1 | | 1.60±0.19 |
| X61123 | Hs.255935 | B-cell translocation gene 1, anti-proliferative | BTG1 | | 1.60±0.28 |
| U48807 | Hs.417962 | dual specificity phosphatase 4 | DUSP4 | | 1.40±0.35 |
| **Transport (7)** | | | | | |
| D16532 | Hs.370422 | very low density lipoprotein receptor | VLDLR | | 2.56±0.31 |
| M20681 | Hs.419240 | solute carrier family 2 (facilitated glucose transporter), member 3 | SLC2A3 | | 2.24±0.39 |
| M21904 | Hs.79748 | solute carrier family 3 ,member 2 | SLC3A2 | | 2.14±0.26 |
| M55531 | Hs.33084 | solute carrier family 2 (facilitated glucose/fructose transporter), member 5 | SLC2A5 | | 1.93±0.44 |
| K03195 | Hs.169902 | solute carrier family 2 (facilitated glucose transporter), member 1 | SLC2A1 | | 1.56±0.13 |
| U53347 | Hs.183556 | solute carrier family 1 (neutral amino acid transporter), member 5 | SLC1A5 | | 1.56±0.13 |
| U32315 | Hs.82240 | syntaxin 3A | STX3A | | 1.43±0.16 |
| **Protein synthesis (3)** | | | | | |
| D12686 | Hs.433750 | eukaryotic translation initiation factor 4 gamma, 1 | | EIF4G1 | 2.26±0.81 |
| U49436 | Hs.433702 | eukaryotic translation initiation factor 5 | | EIF5 | 1.41±0.16 |
| X94754 | Hs.355867 | methionine-tRNA synthetase | | MARS | 1.36±0.25 |
| **Apoptosis (3)** | | | | | |
| S81914 | Hs.76095 | immediate early response 3 | | IER3 | 2.71±0.61 |
| X16706 | Hs.301612 | FOS-like antigen 2 | | FOSL2 | 2.63±0.94 |
| U15174 | Hs.79428 | BCL2/Adenovirus E1B 19kD-interacting protein 3 | | BNIP3 | 2.02±0.31 |
| **Unknown (2)** | | | | | |
| D50911 | Hs.155584 | KIAA0121 gene product | | KIAA0121 | 1.53±0.12 |
| D31884 | Hs.3094 | KIAA0063 gene product | | KIAA0063 | 1.37±0.09 |
| ^a^ Fold change comparing glutathione (GSH) to S-nitrosoglutathione (GSNO)-treated cells is expressed as the mean ± SE (N = 7) | | | | | |
