# Supplementary material for: cGMP-independent nitric oxide signaling and regulation of the cell cycle
Source: BMC Genomics. 2005 Nov 3;6:151. doi: 10.1186/1471-2164-6-151 (PMC1312313; doi:10.1186/1471-2164-6-151)
Supplement: Additional File 2 — Classification of NO•-Downregulated Genes. Complete list of genes downregulated by NO• Genes are classified by function and fold change from control is shown. [file 1471-2164-6-151-S2.doc]

| **Classification of NO·-Downregulated Genes** | | | | | | | |
| --- | --- | --- | --- | --- | --- | --- | --- |
| **GenBank** | **Unigene** | | **Name** | | **Symbol** | | **Fold change** **^a^** |
| **Cell cycle (27)** | | | | | | | |
| U63743 | Hs.69360 | | | kinesin family member 2C | | --- | | | | KIF2C | | --- | | | 0.72±0.10 |
| U83115 | Hs.422550 | | absent in melanoma 1 | | AIM1 | | 0.71±0.07 |
| L13689 | Hs.380403 | | murine leukemia viral (bmi-1) oncogene homolog | | BMI1 | | 0.67±0.09 |
| M34458 | Hs.89497 | | lamin B1 | | LMNB1 | | 0.66±0.16 |
| D63880 | Hs.5719 | | chromosome condensation-related SMC-associated protein 1 | | CNAP1 | | 0.63±0.05 |
| X65550 | Hs.80976 | | antigen identified by monoclonal antibody Ki-67 | | MKI67 | | 0.61±0.09 |
| S78187 | Hs.153752 | | cell division cycle 25B | | CDC25B | | 0.60±0.05 |
| U66838 | Hs.417050 | | cyclin A1 | | CCNA1 | | 0.60±0.12 |
| Z36714 | Hs.1973 | | cyclin F | | CCNF | | 0.60±0.11 |
| D38553 | Hs. 308045 | | barren homolog (Drosophila) | | BRRN1 | | 0.59±0.08 |
| J04088 | Hs.156346 | | topoisomerase (DNA) II alpha | | TOP2A † | | 0.57±0.14 |
| L47276 |  | |  | |  | | 0.42±0.07 |
| X05360 | Hs.334562 | | cell division cycle 2, G1 to S and G2 to M | | CDC2 | | 0.57±0.11 |
| U73379 | Hs.93002 | | ubiquitin-conjugating enzyme E2C | | UBE2C | | 0.54±0.08 |
| U67369 | Hs.73172 | | growth factor independent 1 | | GFI1 | | 0.54±0.06 |
| U30872 | Hs.77204 | | centromere protein F (350/400kD, mitosin) | | CENPF | | 0.53±0.07 |
| Z15005 | Hs.75573 | | centromere protein E (312kD) | | CENPE | | 0.53±0.10 |
| U29343 | Hs.72550 | | hyaluronan-mediated motility receptor (RHAMM) | | HMMR | | 0.51±0.11 |
| U28386 | Hs.159557 | | karyopherin alpha 2 (RAG cohort 1, importin alpha 1) | | KPNA2 | | 0.51±0.05 |
| U05340 | Hs.82906 | | CDC20 (cell division cycle 20, S. cerevisiae, homolog) | | CDC20 | | 0.49±0.07 |
| M86699 | Hs.169840 | | TTK protein kinase | | TTK | | 0.47±0.05 |
| U01038 | Hs.329989 | | polo (Drosophia)-like kinase | | PLK | | 0.44±0.04 |
| D14678 | Hs.20830 | | | Kinesin family member C1 | | --- | | | | KIFC1 | | --- | | | 0.44±0.10 |
| M25753 | Hs.23960 | | cyclin B1 | | CCNB1 | | 0.43±0.07 |
| D38751 | Hs.119324 | | kinesin family member 22 | | | KIF22 | | --- | | | 0.40±0.18 |
| U14518 | Hs.1594 | | centromere protein A (17kD) | | CENPA | | 0.38±0.06 |
| U22376 | Hs.407830 | | v-myb myeloblastosis viral oncogene homolog (avian) | | c-Myb | | 0.30±0.10 |
| U04810 | Hs.171955 | | trophinin associated protein (tastin) | | TROAP | | 0.24±0.03 |
| **Cell proliferation (3)** | | | | | | | |
| D31885 | Hs.75249 | | ADP-ribosylation factor-like 6 interacting protein | | ARL6IP | | 0.79±0.03 |
| X62055 | Hs.63489 | | protein tyrosine phosphatase, non-receptor type 6 | | PTPN6 | | 0.77±0.12 |
| U07802 | Hs.78909 | | zinc finger protein 36, C3H type-like 2 | | ZFP36L2 | | 0.55±0.07 |
| **Signal transduction (3)** | | | | |  | |  |
| V00599 | | Hs.356729 | | FK506-binding protein 1A (12kD) | FKBP1A | | 0.81±0.16 |
| X66113 | | Hs.321234 | | polymyositis/scleroderma autoantigen 2 (100kD) | PMSCL2 | | 0.72±0.05 |
| U76764 | | Hs.3107 | | CD97 antigen | CD97 | | 0.60±0.08 |
| **Inflammation (2)** | | | | | | | |
| L41067 | | Hs.172674 | | nuclear factor of activated T-cells, cytoplasmic, calcineurin-dependent 3 | NFATC3 | | 0.68±0.10 |
| Z48199 | | Hs.82109 | | syndecan 1 | SDC1 | | 0.49±0.12 |
| **Transcription factors (2)** | | | | | | | |
| U75309 | | Hs.96103 | | TAF5 RNA polymerase II,TBP-associated factor, 100 kD | TAF5 | | 0.67±0.07 |
| D87076 | | Hs.397990 | | PHD finger protein 15 | | PHF15 | | --- | | | 0.39±0.08 |
| **Oncogenesis (1)** | | | | | | | |
| U41813 | | Hs.127428 | | homeo box A9 | HOXA9 ^b^ | | 0.76±0.09 |
| U82759 | |  | |  |  | | 0.66±0.14 |
| **Unknown (1)** | | | | | | | |
| D86972 | | Hs.75863 | | KIAA0218 gene product | KIAA0218 | 0.79±0.05 | |
| ^a^ Fold change comparing glutathione (GSH) to S-nitrosoglutathione (GSNO)-treated cells is expressed as the mean ± SE (N = 7)  ^b^ Represented by more than one probe set on the microarray that reached statistical significance; each result is shown | | | | | | | |
