# Supplementary material for: cGMP-independent nitric oxide signaling and regulation of the cell cycle
Source: BMC Genomics. 2005 Nov 3;6:151. doi: 10.1186/1471-2164-6-151 (PMC1312313; doi:10.1186/1471-2164-6-151)
Supplement: Additional File 3 — Confirmation of NO• effects on TNFα, IL-8 and IL-1β (A) NO• up-regulated secreted TNFα, IL-8 and IL-1β protein at 24 h as measured by ELISA. Dibutyryl cAMP (Bt2cAMP) decreased TNFα, increased IL-1β, and had no effect on IL-8. Data are means ± SE of six independent experiments. (B) NO• effect on TNFα, IL-8 and IL-1β mRNA at 6 h as measured by microarray (N = 7) were similar to changes in secreted protein. [file 1471-2164-6-151-S3.ppt]

## Slide 1
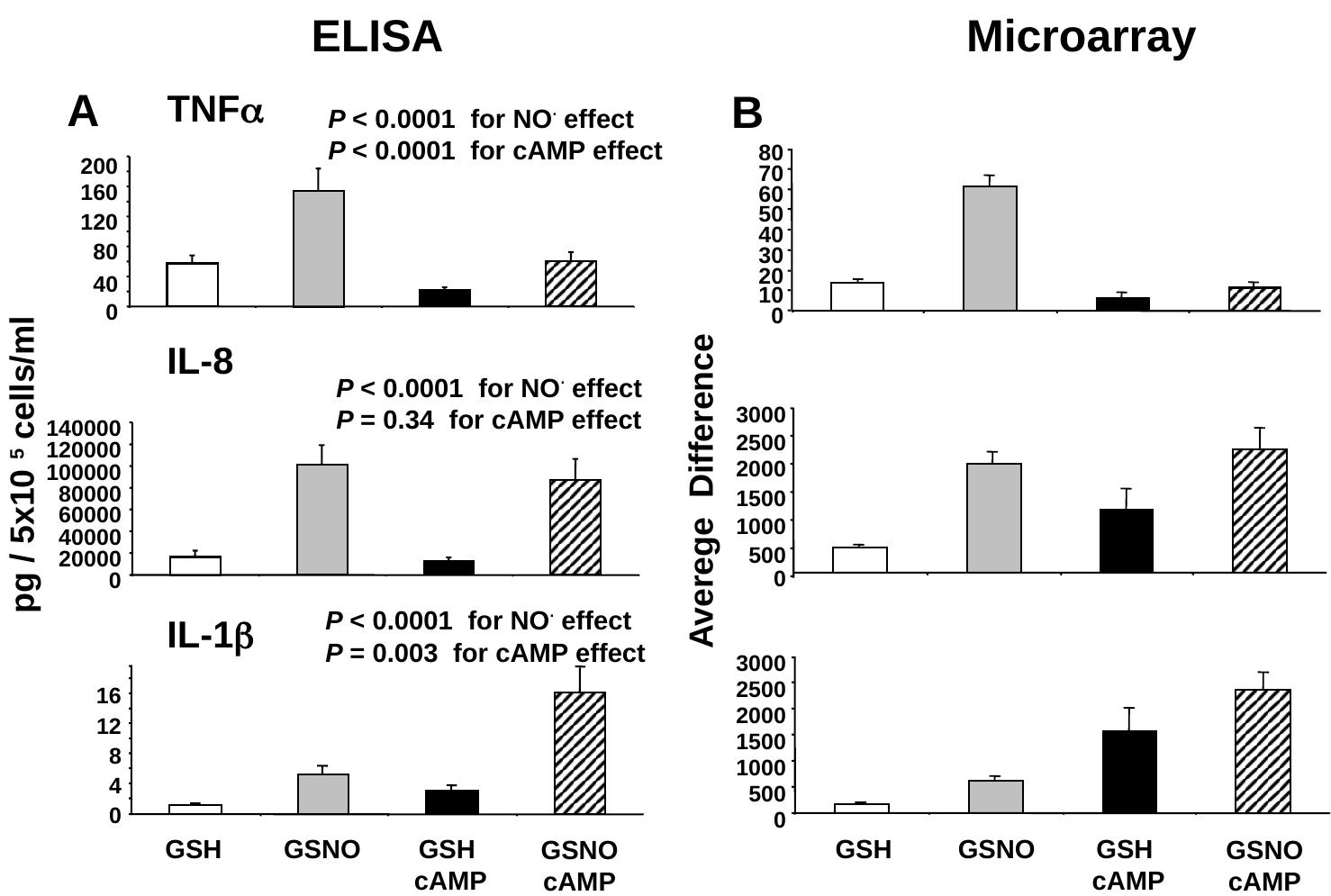

ELISA
TNFα
P < 0.0001 for NO. effect
P < 0.0001 for cAMP effect
200
160
120
80
40
0
IL-8
P < 0.0001 for NO. effect
P = 0.34 for cAMP effect
140000
 pg / 5x10 5 cells/ml
120000
100000
80000
60000
40000
20000
0
P < 0.0001 for NO. effect
P = 0.003 for cAMP effect
IL-1β
16
12
8
4
0
GSH
GSNO
GSH
cAMP
GSNO
cAMP
A
Microarray
80
70
60
50
40
30
20
10
0
3000
2500
2000
Averege Difference
1500
1000
500
0
3000
2500
2000
1500
1000
500
0
GSH
GSNO
GSH
cAMP
GSNO
cAMP
B
