# Supplementary material for: cGMP-independent nitric oxide signaling and regulation of the cell cycle
Source: BMC Genomics. 2005 Nov 3;6:151. doi: 10.1186/1471-2164-6-151 (PMC1312313; doi:10.1186/1471-2164-6-151)
Supplement: Additional File 4 — Classification of cAMP-Regulated Genes. Complete list of genes regulated by cAMP. Genes are classified by function and fold change from control is shown. [file 1471-2164-6-151-S4.doc]

| Classification of cAMP-Regulated Genes | | | |  |  |
| --- | --- | --- | --- | --- | --- |
| **GenBank** | **Unigene** | **Name** | | **Symbol** | **Fold change ^a^** |
| **Metabolism (22)** | | | | | |
| U57721 | Hs.444471 | kynureninase (L-kynurenine hydrolase) | | KYNU | 3.74±0.67 |
| U61836 | Hs.433337 | spermine oxidase | | SMOX | 3.60±0.90 |
| X92521 | Hs.154057 | matrix metalloproteinase 19 | | MMP19 | 2.96±1.22 |
| D89050 | Hs.445299 | oxidised low density lipoprotein (lectin-like) receptor 1 | | OLR1 | 2.63±0.52 |
| U17760 | Hs.436983 | laminin, beta 3 | | LAMB3 | 2.33±0.44 |
| L13943 |  |  | |  | 2.12±0.26 |
| X68285 | Hs.1466 | glycerol kinase | | GK ^b^ | 1.97±0.49 |
| X78711 |  |  | |  | 1.90±0.26 |
| U55853 | Hs.143600 | golgi phosphoprotein 4 | | GOLPH4 | 1.56±0.12 |
| U58046 | Hs.389559 | eukaryotic translation initiation factor 3, subunit 10 (theta, 150/170kD) | | EIF3S10 | 0.70±0.10 |
| L40392 | Hs.197184 | RNA-binding region (RNP1, RRM) containing 7 | | RNPC7 | 0.67±0.08 |
| U43286 | Hs.118725 | selenophosphate synthetase 2 | | SPS2 | 0.66±0.07 |
| D25218 | Hs.71827 | homolog of yeast ribosome biogenesis regulatory protein RRS1 | | RRS1 | 0.65±0.07 |
| J03626 | Hs.2057 | uridine monophosphate synthetase (orotate phosphoribosyl transferase and orotidine-5'-decarboxylase) | | UMPS | 0.63±0.16 |
| U14391 | Hs.437459 | myosin IE | | MYO1E | 0.63±0.11 |
| D10522 | Hs.318603 | myristoylated alanine-rich protein kinase C substrate | | MARCKS | 0.61±0.19 |
| D79993 | Hs.132853 | enthoprotin | | ENTH | 0.58±0.10 |
| D10704 | Hs.77221 | choline kinase | | CHK | 0.55±0.07 |
| L00352 | Hs.213289 | low density lipoprotein receptor (familial hypercholesterolemia) | | LDLR | 0.53±0.09 |
| L36720 | Hs.106880 | bystin-like | | BYSL | 0.51±0.11 |
| M98045 | Hs.754 | folylpolyglutamate synthase | | FPGS | 0.50±0.11 |
| U41387 | Hs.169531 | DEAD/H (Asp-Glu-Ala-Asp/His) box polypeptide 21 | | DDX21 | 0.49±0.19 |
| U08377 | Hs.213739 | splicing factor, arginine/serine-rich 8 (suppressor-of-white-apricot homolog, Drosophila) | | SFRS8 | 0.43±0.09 |
| L05606 | Hs.927 | myosin binding protein H | | MYBPH | 0.37±0.11 |
| **Immune response/Inflammation (21)** | | | | | |
| X04500 | Hs.126256 | Interleukine-1, beta | | IL-1B | 6.64±2.85 |
| M57731 | Hs.75765 | GRO2 oncogene | | GRO2 | 5.74±1.91 |
| D10922 | Hs.99855 | formyl peptide receptor-like 1 | | FPRL1 | 2.86±0.27 |
| AY101192 | Hs.502328 | CD44 antigen (homing function and Indian | | CD44 ^b^ | 2.77±0.55 |
| S66400 |  | blood group system) | |  | 2.64±0.49 |
| L08177 | Hs.784 | Epstein-Barr virus induced gene 2 (lymphocyte-specific G protein-coupled receptor) | | EBI2 | 2.32±0.25 |
| X68090 | Hs.352642 | Fc fragment of IgG, low affinity IIa, receptor for (CD32) | | FCGR2A | 1.94±0.23 |
| X54150 | Hs.193122 | Fc fragment of IgA, receptor for | | FCAR | 1.87±0.40 |
| L38608 | Hs.150693 | activated leucocyte cell adhesion molecule | | ALCAM ^b^ | 1.82±0.31 |
| U30999 |  |  | |  | 1.48±0.24 |
| X66171 | Hs.2605 | CMRF35 leukocyte immunoglobulin-like receptor | | CMRF35 | 1.75±0.39 |
| D79206 | Hs.252189 | syndecan 4 (amphiglycan, ryudocan) | | SDC4 | 1.53±0.26 |
| M94880 | Hs.181244 | major histocompatibility complex, class I, A | | HLA-A ^b^ | 1.52±0.19 |
| D32129 |  |  | |  | 1.42±0.14 |
| M81695 | Hs.385521 | integrin, alpha X (antigen CD11C (p150), alpha polypeptide) | | ITGAX | 1.46±0.23 |
| BC002578 | Hs.381008 | major histocompatibility complex, class I, E | | HLA-E | 1.41±0.16 |
| M61827 | Hs.461934 | sialophorin (gpL115, leukosialin, CD43) | | SPN | 0.70±0.05 |
| M37766 | Hs.243564 | CD48 antigen (B-cell membrane protein) | | CD48 | 0.64±0.12 |
| M63835 | Hs.77424 | Fc fragment of IgG, high affinity Ia, receptor for (CD64) | | FCGR1A | 0.63±0.11 |
| M68874 | Hs.211587 | phospholipase A2, group IVA (cytosolic, calcium-dependent) | | PLA2G4A | 0.62±0.11 |
| M81750 | Hs.153837 | myeloid cell nuclear differentiation antigen | | MNDA | 0.58±0.13 |
| D83597 | Hs.87205 | lymphocyte antigen 64 homolog, radioprotective 105kD (mouse) | | LY64 | 0.57±0.07 |
| M26683 | Hs.303649 | chemokine (C-C motif) ligand 2 | | CCL2 ^b^ | 0.56±0.07 |
| S69738 |  |  | |  | 0.27±0.03 |
| L04270 | Hs.1116 | lymphotoxin beta receptor (TNFR superfamily, member 3) | | LTBR | 0.47±0.16 |
| **Cell cycle (17)** | | | | | |
| X57579 | Hs.28792 | inhibin, beta A (activin A, activin AB alpha polypeptide) | | INHBA | 6.19±2.63 |
| L13391 | Hs.78944 | regulator of G-protein signalling 2, 24kDa | | RGS2 | 4.70±1.41 |
| M54995 | Hs.2164 | pro-platelet basic protein (chemokine (C-X-C motif) ligand 7) | | PPBP | 3.63±2.42 |
| X61123 | Hs.255935 | B-cell translocation gene 1, anti-proliferative | | BTG1 | 3.32±0.52 |
| V01512 | Hs.25647 | v-fos FBJ murine osteosarcoma viral oncogene homolog | | FOS | 2.22±0.33 |
| U48807 | Hs.417962 | dual specificity phosphatase 4 | | DUSP4 | 1.76±0.48 |
| M92843 | Hs.343586 | zinc finger protein 36, C3H type, homolog (mouse) | | ZFP36 | 1.46±0.37 |
| U37022 | Hs.95577 | cyclin-dependent kinase 4 | | CDK4 | 0.76±0.12 |
| U83410 | Hs.82919 | cullin 2 | | CUL2 | 0.76±0.06 |
| X59932 | Hs.77793 | c-src tyrosine kinase | | CSK | 0.68±0.10 |
| M32110 | Hs.15243 | Proliferating-Cell Nucleolar Antigen, 120kDa | | Top of Form  P120Bottom of Form | 0.64±0.07 |
| D21262 | Hs.75337 | nucleolar and coiled-body phosphprotein 1 | | NOLC1 | 0.60±0.20 |
| U67369 | Hs.73172 | growth factor independent 1 | | GFI1 | 0.60±0.06 |
| U66838 | Hs.417050 | cyclin A1 | | CCNA1 | 0.44±0.10 |
| D79994 | Hs.77546 | KIAA0172 protein | | KIAA0172 | 0.41±0.05 |
| L43964 | Hs.25363 | presenilin 2 (Alzheimer disease 4) | | PSEN2 | 0.36±0.09 |
| U22376 | Hs.407830 | v-myb myeloblastosis viral oncogene homolog (avian) | | c-Myb | 0.30±0.14 |
| **Signal transduction (13)** | | | | | |
| L20971 | Hs.188 | phosphodiesterase 4B, cAMP-specific (phosphodiesterase E4 dunce homolog, Drosophila) | | PDE4B | 5.99±1.17 |
| L13740 | Hs.1119 | nuclear receptor subfamily 4, group A, member 1 | | NR4A1 | 4.77±0.60 |
| S68271 | Hs.231975 | cAMP responsive element modulator | | CREM ^b^ | 3.53±0.53 |
| D14826 |  |  | |  | 3.29±0.58 |
| U22431 | Hs.412416 | hypoxia-inducible factor 1, alpha subunit (basic helix-loop-helix transcription factor) | | HIF1A | 2.60±0.43 |
| M74719 | Hs.359289 | transcription factor 4 | | TCF4 | 2.28±0.68 |
| M85169 | Hs.1050 | pleckstrin homology, Sec7 and coiled/coil domains 1(cytohesin 1) | | PSCD1 | 2.17±0.38 |
| D15050 | Hs.380991 | SNF1-like kinase | | SNF1LK | 2.05±0.32 |
| U09937 | Hs.179657 | plasminogen activator, urokinase receptor | | PLAUR | 1.76±0.20 |
| M33336 | Hs.280342 | protein kinase, cAMP-dependent, regulatory, type I, alpha (tissue specific extinguisher 1) | | PRKAR1A | 1.43±0.13 |
| U18062 | Hs.438838 | TAF7 RNA polymerase II, TATA box binding protein (TBP)-associated factor, 55 kD | | TAF7 | 0.72±0.08 |
| L42324 | Hs.88269 | G protein-coupled receptor 18 | | GPR18 | 0.63±0.07 |
| L47345 | Hs.15535 | transcription elongation factor B (SIII), polypeptide 3 (110kD, elongin A) | | TCEB3 | 0.60±0.10 |
| AF006041 | Hs.336916 | death-associated protein 6 | | DAXX | 0.49±0.15 |
| **Cell proliferation (9)** | | | | | |
| M27288 | Hs.248156 | oncostatin M | | OSM | 12.18±2.48 |
| M77349 | Hs.421496 | transforming growth factor, beta-induced, 68kD | | TGFBI | 2.18±0.39 |
| L07592 | Hs.106415 | peroxisome proliferative activated receptor, delta | | PPARD | 1.97±0.57 |
| D87258 | Hs.75111 | protease, serine, 11 (IGF binding) | | PRSS11 | 1.89±0.33 |
| U88964 | Hs.105434 | interferon stimulated gene (20kD) | | ISG20 | 1.62±0.16 |
| X54232 | Hs.328232 | glypican 1 | | GPC1 | 1.28±0.19 |
| D50919 | Hs.370530 | tripartite motif-containing 14 | | TRIM14 | 0.55±0.11 |
| M16750 | Hs.81170 | pim-1 oncogene | | PIM1 | 0.48±0.04 |
| X61118 | Hs.283063 | LIM domain only 2 (rhombotin-like 1) | | LMO2 | 0.44±0.11 |
| **Transcription factor (5)** | | | | | |
| U95740 | Hs.432741 | limkain b1 | | LKAP | 1.63±0.25 |
| L20010 | Hs.83634 | host cell factor C1 (VP16-accessory protein) | | HCFC1 | 0.70±0.05 |
| J03161 | Hs.444086 | serum response factor (c-fos serum response element-binding transcription factor) | | SRF | 0.69±0.04 |
| X98260 | Hs.396817 | zuotin related factor 1 | | ZRF1 | 0.57±0.10 |
| U68019 | Hs.288261 | MAD, mothers against decapentaplegic homolog 3 (Drosophila) | | MADH3 | 0.29±0.05 |
| **Apoptosis (4)** | | | | | |
| X16706 | Hs.220971 | FOS-like antigen 2 | | FOSL2 | 6.12±2.76 |
| S81914 | Hs.76095 | immediate early response 3 | | IER3 | 2.36±0.65 |
| L08246 | Hs.86386 | myeloid cell leukemia sequence 1 (BCL2-related) | | MCL1 | 1.97±0.32 |
| S78085 | Hs.367900 | programmed cell death 2 | | PDCD2 | 0.58±0.24 |
| **GTP binding (4)** | | | | | |
| L22075 | Hs.9691 | guanine nucleotide binding protein (G protein), alpha 13 | | GNA13 | 2.52±0.44 |
| M63904 | Hs.73797 | guanine nucleotide binding protein (G protein), alpha 15 (Gq class) | | GNA15 | 2.23±0.63 |
| M57763 | Hs.89474 | ADP-ribosylation factor 6 | | ARF6 | 0.70±0.07 |
| U59878 | Hs.32217 | RAB32, member RAS oncogene family | | RAB32 | 0.64±0.17 |
| **MAPKKK cascade (3)** | | | | | |
| M62505 | Hs.2161 | | complement component 5 receptor 1 (C5a ligand) | C5R1 | 1.87±0.71 |
| D87116 | Hs.180533 | | mitogen-activated protein kinase kinase 3 | MAP2K3 | 1.49±0.19 |
| M15169 | Hs.2551 | | adrenergic, beta-2-, receptor, surface | ADRB2 | 0.57±0.04 |
| **Steroid biogenesis (3)** | | | | | |
| M11058 | Hs.11899 | | 3-hydroxy-3-methylglutaryl-Coenzyme A reductase | HMGCR | 0.61±0.13 |
| U60205 | Hs.393239 | | sterol-C4-methyl oxidase-like | SC4MOL | 0.57±0.11 |
| D13643 | Hs.75616 | | 24-dehydrocholesterol reductase | DHCR24 | 0.56±0.08 |
| **Ras GTPase activator activity (2)** | | | | | |
| D29640 | Hs.1742 | | IQ motif containing GTPase activating protein 1 | IQGAP1 | 1.25±0.19 |
| U51903 | Hs.373980 | | IQ motif containing GTPase activating protein 2 | IQGAP2 | 0.72±0.05 |
| **Unknown (3)** | | | | | |
| D31884 | Hs.3094 | | KIAA0063 gene product | KIAA0063 | 1.93±0.19 |
| D13645 | Hs.443866 | | KIAA0020 gene product | KIAA0020 | 0.59±0.15 |
| D87716 | Hs.90315 | | KIAA0007 protein | KIAA0007 | 0.43±0.11 |
| ^a^ Fold change comparing glutathione (GSH) to dibutryl cAMP (Bt_2_cAMP) plus GSH-treated cells is expressed as the mean ± SE (N = 7)  ^b^ Represented by more than one probe set on the microarray that reached statistical significance; each result is shown | | | | | |

# 
