# Supplementary material for: cGMP-independent nitric oxide signaling and regulation of the cell cycle
Source: BMC Genomics. 2005 Nov 3;6:151. doi: 10.1186/1471-2164-6-151 (PMC1312313; doi:10.1186/1471-2164-6-151)
Supplement: Additional File 5 — RT-PCR Primers and Probes. List of genes tested by RT-PCR including the sequence of primers and probes used in the assays. [file 1471-2164-6-151-S5.doc]

# RT-PCR Primers and Probes

| **GenBank** | **Symbol** | **Forward primer** | **Reverse primer** | **Probe** |
| --- | --- | --- | --- | --- |
| X06985 | HMOX1 | AGCCCTGCCCTTCAGCAT | TTGAGACAGCTGCCACATTAGG | comp-TGTCTTCCAGGCTCTGCTGCAGGAACT |
| L19871 | ATF3 | TTTGCCATCCAGAACAAGCA | GGGTCTGTCGCTGACAGTGA | ACCGGATGTCCTCTGCGCTGGA |
| M57731 | GRO2 | CAAGAACATCCAAAGTGTGA | GCCCATTCTTGAGTGTGGCTAT | CCCACTGCGCCCAAACCGAAGT |
| J04111 | JUN | GGGTTGACTGGTAGCAGATAAGTGTT | CTTCAGCCACACTCAGTGCAA | comp-CTGAGCCCTTATCCAGCCCGAGCT |
| D14874 | ADM | CGCCCATGGTACAAGGAATAG | ACCGCTCGGGAAGTCCTT | CGCAAGCATCCCGCTGGTGC |
| M27288 | OSM | CCCTGCCTCGGATGCTTT | CTGAGTGCATGAAGCGATGGT | comp-AGGAACCTGCAGCCCTCCAGCTTG |
| X99920 | S100A13 | AGCCTCAGCGTCAACGAGTT | CATCAAGAGAGCCCACATCCTT | CCCAGCAGTTGCCCCATCTGCT |
| U57721 | KYNU | TCCATTGGGATCCTAGCTGTTT | AATGCGCTGCACTGTGTCA | AATGGAGCCTTCATCTCTTGAGCTGCC |
| U09579 | CDKN1A | GCAGACCAGCATGACAGATTTC | GCGGATTAGGGCTTCCTCTT | CACTCCAAACGCCGGCTGATCTTC |
| S49592 | E2F1 | AGCAGATGGTTATGGTGATCAAAG | GGAGATCTGAAAGTTCTCCGAAGA | CTCCTGAGACCCAGCTCCAAGCCG |
| U66838 | CCNA1 | ATGGCATTTGAGGATGTGTATGAA | CTGTGTTGAAATCCAGCAGGAA | CCGGCACACTCAAGTCAGACCTGC |
| Z15005 | CENPE | TCGAGATAGCAAGTTAACACGAATTC | ACTGGCAAACTGGAGAGCAGTAA | AGACACGTATTATCTGCACAATTACTCCAG |
| U01038 | PLK | GGATCACACCAAGCTCATCTTG | CCCGCTTCTCGTCGATGT | CCCACTGATGGCAGCCGTGACC |
| M25753 | CCNB1 | CCCTGCTGCAACCTCCAA | AGTTGTTCACTGACTTTGTTACCAATG | CCGGACTGAGGCCAAGAACAGCTC |
| U22376 | c-Myb | TCGAGTGCATCAACCCTGAA | TAACTTTTCCTTTATCTGCTCTGACAAG | comp-CTCAGACCCAACAGTCAGAGGATTTTCAAAGG |

# The primers and probes of IL8 (M28130), IL1B (X04500), and TNF (X02910) were purchased from ABI
