# Supplementary material for: cGMP-independent nitric oxide signaling and regulation of the cell cycle
Source: BMC Genomics. 2005 Nov 3;6:151. doi: 10.1186/1471-2164-6-151 (PMC1312313; doi:10.1186/1471-2164-6-151)
Supplement: Additional File 6 — Electrophoretic Mobility Shift Assay (EMSA) Probes. List of genes from which E2F and CDE/CHR promoter sequences were derived for testing by electrophoretic mobility shift assay (EMSA). For each gene, the EMSA probe sequence is shown. [file 1471-2164-6-151-S6.doc]

# Electrophoretic Mobility Shift Assay (EMSA) Probes

| E2F1 | 5’-CGTGGCTCTTTCGCGGCAAAAAGGA-3’ |
| --- | --- |
| Mut E2F1 | 5’-CGTGGCTCTTTCGATGCAAAAAGGA-3’ |
| CDC2 | 5’-TTAGCGCGCTGAGTTTGAAACT-3’ |
| CDC6 | 5’-CCGGGCTTTGGCGGGAGGTGGG-3’ |
| PLK | 5’-GTTCCCAGCGCCGCGTTTGAATTC-3’ |
| Mut PLK | 5’-GTTCCAAGCTTCGCGGTCCACTTC-3’ |
| CDC2 | 5’-TTAGCGCGCTGAGTTTGAAACT-3’ |
| Cyclin A | 5’-GTAGTCGCGGGATACTTGAACTG-3’ |
| Cyclin B | 5’-GAGCAGTGCGGGGTTTAAATCT-3’ |
